# Investigation of a cross-border case of Lassa fever in West Africa

**DOI:** 10.1186/s12879-019-4240-8

**Published:** 2019-07-10

**Authors:** Mory Keïta, Georges Alfred Kizerbo, Lorenzo Subissi, Fodé Amara Traoré, Ahmadou Doré, Mohamed Fode Camara, Ahmadou Barry, Raymond Pallawo, Mamadou Oury Baldé, Nfaly Magassouba, Mamoudou Harouna Djingarey, Ibrahima Socé Fall

**Affiliations:** 1 Organisation Mondiale de la Santé - Bureau Régional de l’Afrique, BP: 06, Cité du Djoué, Brazzaville, Congo; 2 Organisation Mondiale de la Santé - Bureau de Pays de la Guinée, BP: 817, Immeuble BAH, Quartier Cameroun, Conakry, Guinea; 3 Sciensano, 1050 Ixelles, Rue Juliette Wytsman 14, Brussels, Belgium; 4 Université Gamal Abdel Nasser, Faculté des Sciences et Techniques de la Santé, BP: 1147, Commune de Dixinn, Route de Donka, Quartier Landréah, Conakry, Guinea; 5 Université Gamal Abdel Nasser, Laboratoire National des Fièvres Hémorragiques de Guinée, Carrefour Nongo-Conteyah, Conakry, Guinea; 6 Ministère de la Santé, BP: 585, Boulevard du commerce - Almamya Kaloum, Conakry, Guinea

**Keywords:** Lassa fever, Lassa virus, Cross-border, Outbreak investigation, Case report, Guinea, Liberia, West Africa

## Abstract

**Background:**

Infectious disease prevention and control strategies require a coordinated, transnational approach. To establish core capacities of the International Health Regulations (IHR), the World Health Organization (WHO) developed the Integrated Diseases Surveillance and Response (IDSR) strategy. Epidemic-prone Lassa fever, caused by Lassa virus, is an endemic disease in the West African countries of Ghana, Guinea, Mali, Benin, Liberia, Sierra Leone, Togo and Nigeria. It’s one of the major public health threats in these countries. Here it is reported an epidemiological investigation of a cross-border case of Lassa fever, which demonstrated the importance of strengthened capacities of IHR and IDSR.

**Case presentation:**

On January 9th, 2018 a 35-year-old Guinean woman with fever, neck pain, body pain, and vomiting went to a hospital in Ganta, Liberia. Over the course of her illness, the case visited various health care facilities in both Liberia and Guinea. A sample collected on January 10th was tested positive for Lassa virus by RT-PCR in a Liberian laboratory.

The Guinean Ministry of Health (MoH) was officially informed by WHO Country Office for Guinea and for Liberia.

**Conclusion:**

This case report revealed how an epidemic-prone disease such as Lassa fever can rapidly spread across land borders and how such threat can be quickly controlled with communication and collaboration within the IHR framework.

## Background

Infectious disease threats require effective transnational approaches for prevention, detection, and response [1–5]. The International Health Regulations (IHR), proposed by the World Health Organization (WHO) and signed by UN countries in 2005, require that all countries around the world commit to develop and maintain core public health capacities needed to detect, diagnose, report and respond to public health threat [6].

Lassa virus (LASV) is a single stranded RNA virus of the *Arenaviridae* family [7] that causes Lassa fever. The literature reports that 80% of Lassa fever cases can remain asymptomatic especially in an endemic area [8]. Lassa fever can sometimes manifest as a viral hemorrhagic fever, and it is difficult to recognize and detect rapidly. No vaccine treatment is available [9]; ribavirin is the antiviral treatment of choice and it is reasonably effective if given early on in the course of clinical illness. Lassa fever has a case fatality rate up to 70% when left untreated [10]. LASV is endemic in West Africa, with an estimated tens of thousands of cases annually [11, 12], and the number of sporadic cases outside the endemic regions within and outside Africa is increasing as a result of international travel [13]. This translates into an urgent need to train healthcare providers on LASV, its life cycle and its clinical manifestations [14]. Recently, the Guinean Ministry of Health (MoH) declared an outbreak after a patient from forested Guinea tested positive for LASV by reverse transcriptase – polymerase chain reaction (RT-PCR) [15].

This case report describes an investigation of a patient with laboratory confirmed Lassa fever. This cross-border investigation was a successful example of cooperation between two West African countries, within the framework of Integrated Diseases Surveillance and Response (IDSR) and IHR, successfully implemented in the field.

## Case presentation

**Fig. 1:**
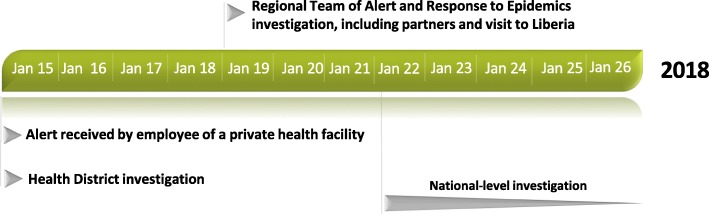
Timeline of the investigation activities carried out by the health district, the health region and the national level

On January 15th, 2018, Guinean health authorities were informed of a confirmed Lassa fever case by the WHO Country Office for Guinea, after being informed by their Liberian counterparts. The case came from the Diecke area, at the border between Guinea and Liberia. Following this notification, a first investigation was undertaken by the District Health Management Team of Yomou in the following 24 h. A second investigation was conducted by Nzerekore’s Regional Team of Alert and Response to Epidemics and relevant partners on January 19th, 2018. During this investigation, the Guinean team visited Liberia and held a meeting with staff of the health facility where the case deceased. Because of the public health importance of Lassa Fever, and the need for effective risk assessment, a third and in-depth retrospective investigation was led by the national level (Fig. 1).

**Fig. 2:**
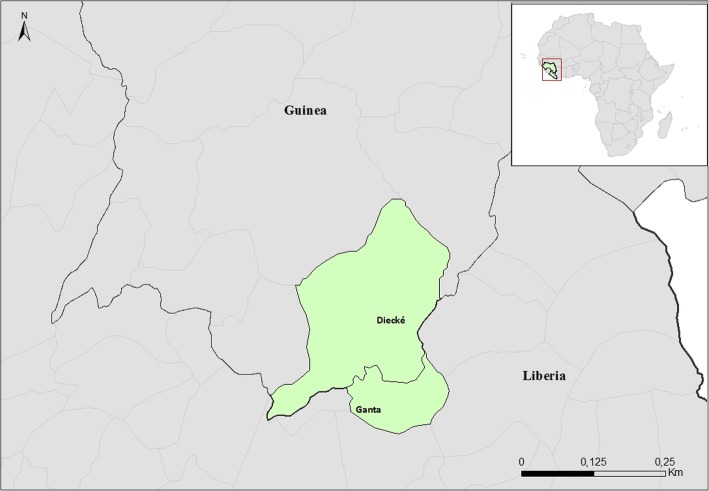
Map of the health areas (in green) visited by the case during her illness, ie Diecke in Guinea, and Ganta in Liberia. *(Source: Map made in the software ArcGIS 10.2 with shapes files from* *diva-gis.org**)*

The case-patient was a 35-year-old Guinean woman who lived and worked in Diecké, Health District of Yomou, Guinea and she regularly crossed the border to the town of Ganta, Liberia (Fig. 2). On January 2nd, 2018 she visited “Hospital 1”, declaring she experienced chills, fever and anorexia over the previous week. A rapid diagnostic test (RDT) for malaria was positive and she received ceftriaxone, quinine and supportive treatment. On January 4th, 2018, she went to the same hospital for a second consultation for physical asthenia; she had low blood pressure and was given supportive treatment. On January 5th, 2018, she made a third consultation at “Hospital 2”, for fever, headache, anorexia, fluid stools, abdominal pain, and physical asthenia evolving over 2 days. The malaria RDT was again positive. She received metronidazole and arthmeter-lumefantrine treatment. On January 9th, 2018, the patient refused to go to the District Hospital, after she had a fourth consultation at “Hospital 1” for fluid stool, vomiting, stiffness, fever, and physical asthenia. She did not get treatment, but she asked for sick leave to go to Liberia.

On January 9th, 2018 she was admitted to “Hospital 3” in Ganta (Liberia), for fever, neck pain, body pain, and vomiting. She was treated first for typhoid before Lassa virus infection was suspected. On January 10th, 2018, a blood sample was collected and was sent to the Liberian National Reference Laboratory, where it tested positive for Lassa virus by reverse transcription polymerase chain reaction (RT-PCR). She died on January 11th, 2018 and a safe and dignified burial was performed in Liberia by the hospital’s burial team. Twenty-eight contacts, including 22 healthcare workers, were identified in Guinea and 28 contacts, including 16 healthcare workers and 12 family members, were identified in Liberia. Almost all contacts were closely monitored: their temperatures were taken at least once a day. This was done until the end of the 21 days’ follow-up period, which corresponds to the maximum incubation period for LASV. On January 18th, 2018, 2 of her contacts in Liberia became symptomatic, but both tested negative for Lassa virus by RT-PCR. The active search of febrile cases in health facilities and analysis of community death data showed no increase in reporting.

## Discussion and conclusions

**Fig. 3:**
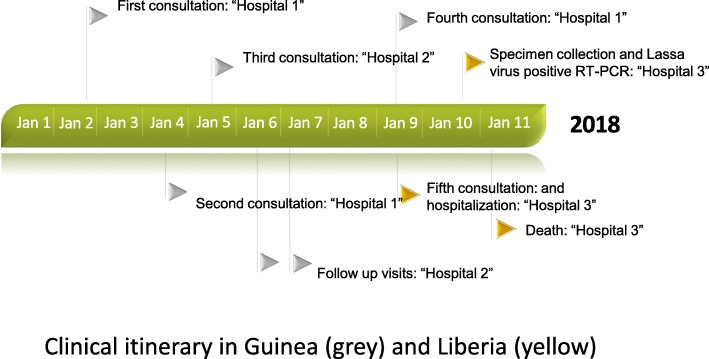
Clinical itinerary of a cross-border Lassa fever case between Guinea and Liberia, January 2018

Here it is reported a fatal case of Lassa fever at the border between Guinea and Liberia. The case investigation highlighted difficulties and delays in the diagnosis of the case-patient in an endemic area of Lassa virus (Fig. 3). Lassa fever diagnostic is challenging based on clinical signs and symptoms alone. Early-stage Lassa fever presents similarly to other febrile illnesses (e.g. malaria, typhoid fever, Ebola virus disease), and it is often suspected only after development of hemorrhagic symptoms in the late stages of the disease [15].

During the in-depth investigation, a list of contacts of the case-patient was conducted as well as a search for cases with clinical presentations compatible with Lassa fever. Medical records were checked for any abnormal increase in reported febrile illness in health care facilities within 5 km to the hospitals visited by the patient. The database of events’ alerts from community surveillance officers looking for suspect deaths or abnormal increases in the number of reported deaths was analyzed.

The seroprevalence of antibodies for Lassa virus in the local population is estimated to be 13% [16, 17]. While in Liberia outbreaks of Lassa fever are declared regularly, no outbreak has been reported in the last decade in Guinea [18]. This may be due to lack of clinical awareness of the endemicity of the disease, or the lack of laboratory capacities in Guinea. Interestingly, on February 3rd, 2019, a year after our investigation, the Guinean MoH declared the first Lassa fever outbreak since decades, after one patient tested positive for LASV by RT-PCR.

From January 1st, 2017 to January 23rd, 2018, Liberian health authorities reported 91 suspected cases from six counties, including Nimba, at the border with Guinea [19]. Thirty-three of these cases were laboratory confirmed, including 15 deaths (case fatality rate for confirmed cases = 45.4%). The risk assessment revealed a stable trend in the number of cases in Liberia.

Active case finding, contact tracing, laboratory support and risk communication (both in the community and through training of health care workers) were the key activities undertaken in Liberia and some areas in Guinea bordering Nimba county. This also demonstrated the necessity to integrate private healthcare centers in IDSR by exhaustive identification of these centers, support for healthcare worker training and facility materials, and weekly surveillance data collection.

For this investigation, sequencing data were not available. These would have proven helpful for understanding the source of transmission and subsequent transmission patterns [20]. Our investigation could therefore not conclude if the exposure occurred in Guinea or Liberia.

The efficiency of the implementation of IDSR in Guinea allowed a first epidemiological investigation of the alert within 24 h, as recommended. The subsequent investigations established public health interventions for Lassa Fever outbreak control in Guinea. Similar public health interventions were implemented in Liberia. The IDSR guidelines from the WHO Regional Office for Africa [21] do not report the criteria for assigning such case to a specific country, and we recommend that future revisions of IDSR should accommodate and clarify cross-border cases like this into account.

## Data Availability

N/A.

## Abbreviations

IDSR: Integrated diseases surveillance and response
IHR: International Health Regulation
LASV: Lassa virus
MoH: Ministry of Health
RDT: Rapid diagnosis test
RT-PCR: Reverse transcriptase polymerase chain reaction
WHO: World Health Organization
